# G Protein-Coupled Receptors as Potential Intercellular Communication Mediators in Trypanosomatidae

**DOI:** 10.3389/fcimb.2022.812848

**Published:** 2022-05-16

**Authors:** Emilia Díaz, Anthony Febres, Michelle Giammarresi, Adrian Silva, Oriana Vanegas, Carlos Gomes, Alicia Ponte-Sucre

**Affiliations:** ^1^ Laboratory of Molecular Physiology, Institute of Experimental Medicine, School of Medicine Luis Razetti, Faculty of Medicine, Universidad Central de Venezuela, Caracas, Venezuela; ^2^ Section of Infectious Diseases, Baylor College of Medicine, TX, United States; ^3^ Pediatric Gastroenterology, University of Iowa, Iowa City, IA, United States; ^4^ Royal Berkshire NHS, Foundation Trust, Light House Lab, Bracknell, United Kingdom; ^5^ Medical Mission Institute, Würzburg, Germany

**Keywords:** GPCR receptors, RAMPs, leishmania, trypanosomatidae, cell-cell communication, skin neuropeptides, stress responses

## Abstract

Detection and transduction of environmental signals, constitute a prerequisite for successful parasite invasion; i.e., *Leishmania* transmission, survival, pathogenesis and disease manifestation and dissemination, with diverse molecules functioning as inter-cellular signaling ligands. Receptors [i.e., G protein-coupled receptors (GPCRs)] and their associated transduction mechanisms, well conserved through evolution, specialize in this function. However, canonical GPCR-related signal transduction systems have not been described in *Leishmania*, although orthologs, with reduced domains and function, have been identified in Trypanosomatidae. These inter-cellular communication means seem to be essential for multicellular and unicellular organism’s survival. GPCRs are flexible in their molecular architecture and may interact with the so-called receptor activity-modifying proteins (RAMPs), which modulate their function, changing GPCRs pharmacology, acting as chaperones and regulating signaling and/or trafficking in a receptor-dependent manner. In the skin, vasoactive- and neuro- peptides released in response to the noxious stimuli represented by the insect bite may trigger parasite physiological responses, for example, chemotaxis. For instance, in *Leishmania* (*V*.) *braziliensis*, sensory [Substance P, SP, chemoattractant] and autonomic [Vasoactive Intestinal Peptide, VIP, and Neuropeptide Y, NPY, chemorepellent] neuropeptides at physiological levels stimulate *in vitro* effects on parasite taxis. VIP and NPY chemotactic effects are impaired by their corresponding receptor antagonists, suggesting that the stimulated responses might be mediated by putative GPCRs (with essential conserved receptor domains); the effect of SP is blocked by [(D-Pro 2, D-Trp7,9]-Substance P (10^-6^ M)] suggesting that it might be mediated by neurokinin-1 transmembrane receptors. Additionally, vasoactive molecules like Calcitonin Gene-Related Peptide [CGRP] and Adrenomedullin [AM], exert a chemorepellent effect and increase the expression of a 24 kDa band recognized in western blot analysis by (human-)-RAMP-2 antibodies. *In-silico* search oriented towards GPCRs-like receptors and signaling cascades detected a RAMP-2-aligned sequence corresponding to *Leishmania* folylpolyglutamate synthase and a RAMP-3 aligned protein, a hypothetical *Leishmania* protein with yet unknown function, suggesting that in *Leishmania*, CGRP and AM activities may be modulated by RAMP- (-2) and (-3) homologs. The possible presence of proteins and molecules potentially involved in GPCRs cascades, i.e., RAMPs, signpost conservation of ancient signaling systems associated with responses, fundamental for cell survival, (i.e., taxis and migration) and may constitute an open field for description of pharmacophores against *Leishmania* parasites.

## Is There a Sensory Function in *LEISHMANIA*? A Brief Introduction

**Figure 1:**
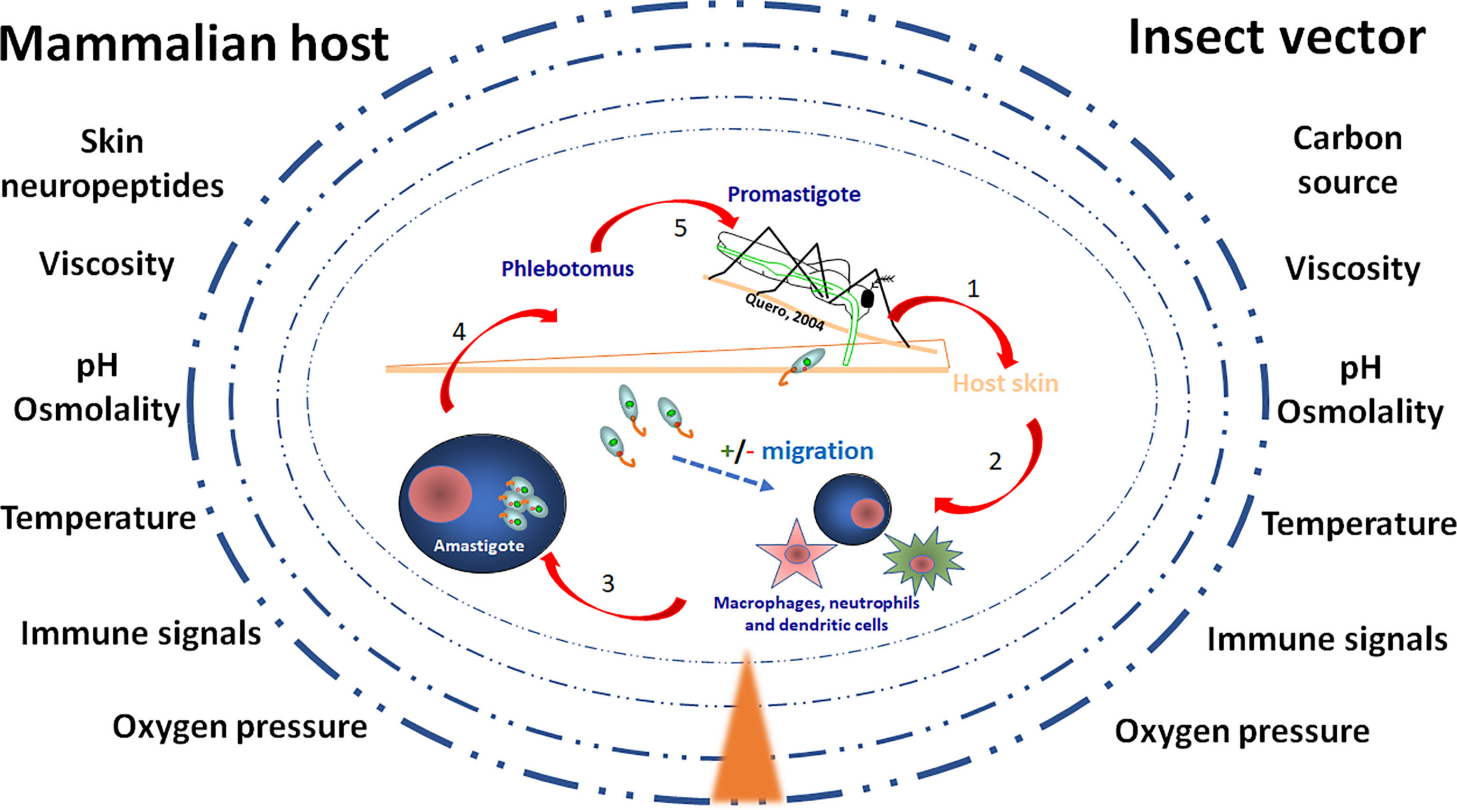
The environment of *Leishmania* in the vector and the host skin. The figure illustrates the gradient (▲) of environmental compounds and signals to which *Leishmania* parasites are exposed during their life cycle. Once the vector bites and *Leishmania* parasites enter the skin, unique mammalian host signals constituted by various neuropeptides and neuropeptide-like mediators are released, potentially modulating migration of parasites towards or against their host cells, being macrophages, neutrophiles and dendritic cells. After a blood meal by the female sand fly vector parasites reach the gut microenvironment where growth and development as morphologically distinct forms of extracellular promastigotes is also subjected to unique signals. The challenges to which the parasites are exposed include carbon nutrients, immune signals, skin neuropeptides, osmolality, oxygen pressure, pH, temperature, viscosity.

Fluctuating levels of chemicals, nutrients, pressure and temperature (see **Figure 1**) constitute dynamic and stressful conditions to which *Leishmania* parasites are normally exposed both in the vertebrate host and in the insect vector, and especially when moving between them. To succeed within the hostile landscape represented by their hosts, these parasites must have sensing capabilities to detect external environmental changes, and cell-cell interaction processes -originated in ancient times- are key steps for successful infection (Zuzarte-Luís and Mota, 2018). Even more, transition between hosts, with different physiological *milieu* is normally sudden and requires rapid molecular and cellular reprogramming. For example, temperature and pH changes are key determinant elements during the life cycle of *Leishmania*; thus, communication between host and parasite must be constant and each influence the response of its partner. In fact, the parasite releases molecules that, as we will discuss afterwards, influence the host response (Zuzarte-Luís and Mota, 2018). 

At physiological levels these organisms then adjust, the expression of specific genes, the activity of certain proteins, or change cell cycle progression, in response to environmental stressful signals oscillations. However, signaling pathways used by Trypanosomatidae (order Kinetoplastidae), to perceive “what’s out there”, and their transformation into behavioral responses, have remained elusive, partially due to the fact that molecules involved in these pathways differ in the protozoan parasites (Gould and de Koning, 2011) when compared to higher eukaryotes.

In fact, in these parasites, genome size has been reduced; there is a lack of gene regulation at the transcription level; and genes are transcribed in a polycistronic way, not constituting operons of functionally related and jointly regulated genes (Grünebast and Clos, 2020). This means that Trypanosomatidae cannot rapidly upregulate the needed genes to adapt to environmental adversity. Still, they express constitutive genetic variability, traduced in practical terms in aneuploidy (meaning that chromosomes may be supernumerary or not present), as well as the capability to select specific haplotypes that may be fundamental for their survival; both behaviors represent an advantage that permits their physiological adaptation, although they may also represent a potential loss of genetic heterogeneity. This behavior, beautifully evaluated in diverse *Leishmania* species, including *Leishmania (L.) donovani* (Prieto Barja et al., 2017) means that Trypanosomatidae retain flexibility to adapt as a population, to diverse environmental challenges (Prieto Barja et al., 2017; Grünebast and Clos, 2020).

Furthermore, although the genomes of Trypanosomatidae have been sequenced (Berriman et al., 2005; El-Sayed et al., 2005; Ivens et al., 2005), most membrane protein types mediating environment sensation in eukaryotes, such as G-protein-coupled receptors (GPCRs), heterotrimeric G-proteins or receptor tyrosine kinases, seem not to be present in these unicellular eukaryotes (Landfear and Zilberstein, 2019), at least as homolog proteins to those present in higher eukaryotes. Additionally, signaling mechanisms mediated by orthologs remain to be fully described in these organisms. How then these pathogens detect and respond to stress and to environmental changes and support successful parasitism remains an open question (Landfear and Zilberstein, 2019). Of note, some hints have appeared, at least in apicomplexan parasites (Pereira and García, 2021). In a gene-edited *Plasmodium falciparum* strain, knocked out for *PfSR25*, a putative GPCR [PfSR25] has been described (Moraes et al., 2017). This putative GPCR seems to be very similar in all *Plasmodium* species, but appears not to have homologous protein in humans. PfSR25 seems to be an ionic sensor for detecting extracellular K^+^ concentration changes common during the egress/invasion process.

Protozoan parasite cyclic nucleotide signaling systems must for sure exist, sharing characteristics, but with striking differences, with their mammalian hosts. This is the case for example of the canonical building blocks of cascades like cyclase, phosphodiesterase and nucleotide-specific protein kinases. These mechanisms although expressed in these ancient parasites, have an activation mode and action that has not been completely elucidated. Particularly, GPCRs seem not to be present in their genome, at least in their canonical form (Tagoe et al., 2015).

But, could it be that G-protein signaling pathways are entirely absent in Trypanosomatidae?

To analyze in deep these fundamental questions, herein we examined the evolutionary perspective of signaling, followed by a brief description of the G-protein signaling scheme, including what is known in these parasites about transduction systems. The final discussion address what has been described in *Leishmania*, as well as our contribution to this theme, especially regarding the so-called receptor associated modifying proteins (RAMPs).

Our aim has been to explore function and roles GPCRs’ and their associated molecules may have in environmental sensing in Trypanosomatidae. Underscoring the way GPCRs’ work in these organisms may constitute an indicator of the development of receptor sensory function in these parasites and their role in adaptive evolution. This understanding of their differential structure, and function during pathogenesis, may additionally represent a challenging opportunity for the identification of unique targets in the search of selective pharmacological inhibitors against the invasion processes.

## Evolutionary Perspective of Signaling

Compared with multicellular, unicellular forms of living organisms express limited number of genes and proteins. However, both types of life forms process surrounding signals and transform them into a significant behavior. In fact, signal transduction networks, due to their dynamic features, are constantly under pressure and selection, and therefore in evolution. A reliable signal transduction system needs specificity and amplification, even at the unicellular level, although signaling mechanisms may not be as complex as in multicellular organisms. Correct stimuli processing needs sophisticated networks (van Albada and ten Wolde, 2007) and mechanisms to amplify signals reaching transducers (positive feedback, or cooperative binding of intracellular signaling molecules to receptors, or receptor molecules interactions) (Bray et al., 1998; Nair et al., 2019). This means that even for primitive eukaryotes such as Trypanosomatidae, a decisive evolution stage must include successful signal amplification and effective inter-cellular communication, to guarantee survival. This group of organisms is extremely diverse and adaptable to environmental conditions, and being considered as a divergent evolutionary lineage, they cannot be classified as a “classical” model for eukaryotes (Maslov et al., 2019); this description includes also signal transductions systems.

Detection of external stimuli is conducted by dynamic interactions between cell surface receptors and extracellular ligands (Chen et al., 2017). In fact, the receptor-ligand concept is an ancient duo that exemplifies how in biology, when a series of causally interacting steps producing one or more effects works successfully, it is used repeatedly, either modulated or sophisticated, but in principle, always the same. Recent advances in the comprehension of this relationship include the use of computational models that realistically simulate the binding process between multi-specific ligands and membrane receptors on cell surfaces (Chen et al., 2017), simplifying receptor and binding sites in a multi-specific ligand system into a rigid structure. In this way, it is possible to test the relation between overall binding of multi-specific ligands and the affinity of their cognate binding site (Chen et al., 2017).

Evolution incorporates complexity through an increasing number of processes that have to be controlled to work in a coordinate way. The end result, a meaningful behavior. How evolution of the receptor–ligand pair occurred is beyond the scope of this contribution; we invite the readers to revise the publication of Nair et al., 2019, and the references within to have a brief overview of the theme.

Cellular and morphological diversity arise from the function of conserved pathways; however, path specificity needs cell type, structural, biochemical, and biophysical characteristics as well as a profound cross-talk between various signaling pathways (Portilla-Martínez et al., 2020). In other words, specificity should have for sure evolved in the structure of both ligand and receptor in a continuous way (Moyle et al., 1994), and probably in parallel [receptors arose devoid of ligands, and receptor–ligand interaction occurred later in evolution (Laudet, 1997)], and not necessarily in an interactive way (Grandchamp and Monget, 2020). Additionally, since ancient times, similar messengers seem to be used when environmental signals reach the cells. This suggests that variations in the magnitude and spatiotemporal location of messengers, their receptors and effectors, brought-in the concept of diversity (Gerhart and Kirschner, 2007), so fundamental in biology and in evolution. This also means that evolution of the receptor-ligand pair concept “encouraged” concomitant development of information transmission processes. As a consequence, several interacting partners arose, networking in multiple pathways, adding intricacy to the system and enriching the complexity of communication potential. The understanding of this complex process is fundamental, not only for the comprehension of the pathology of the disease, but also for the successful design of selective drugs against the parasite, and even in this era of modern molecular technology and next generation sequencing platforms, still represent a huge challenge.

## G Protein Related Receptors

When changes in the environment [mechanical (mechanotransduction), electrical (electrotransduction), or chemical (chemotransduction, the most common)] occur, cells respond with physiological or behavioral responses needed to preserve cellular homeostasis.

Upon these changes, cell receptors transduce the extracellular signals (Nair et al., 2019) and trigger cell networks that activate reprogramming of biochemical, genetic, and structural processes through webs that amplify signaling and ends up in the needed physiological response. Thus, ligand binding (the signal) to its receptor, (1) promotes receptor conformational changes, (2) activate well-controlled reactions through the coordinated work of signaling intermediates (second messengers), and (3) transduce the message from receptors to effector systems and produce a cell response (Kaiser, 2019).

Signal transduction does not mean a linear sequential activation cascade of signaling molecules. On the contrary, it should rather be defined as an “orchestra” in which intracellular signaling nodes (web) establish the communication mechanism. Hence, one stimulus triggers downstream signaling but, as normally occurs, ligand-bound receptors recruit connectors and engage assorted signaling intermediates that transmit the signal further through various additional second messengers. The crosstalk amongst them integrates the information, and transmit it to cytosol and/or nucleus target molecules to end up in the triggering of effector functions (Nair et al., 2019).

Cell-surface and intracellular receptors constitute main categories of receptors; cell-surface receptors spanning the plasma membrane and characterized by an extracellular ligand binding domain, a hydrophobic transmembrane domain and a cytoplasmic domain. Further classifications include the so-called G-protein coupled receptors, ionotropic receptors, and receptor tyrosine kinases (Nair et al., 2019). Herein we concentrate on G-protein coupled receptors.

The G-protein coupled receptors (GPCRs) are transmembrane proteins linked to intracellular signaling molecules such as GTP-binding proteins and protein kinases (Thomason et al., 2002). These signaling proteins are defined as multi-protein complexes that regulate in a strictly controlled way signaling location, duration and specificity. For example, minimal changes in ligand concentration produce a chemotactic response that is intracellularly amplified even with very small changes in overall receptor occupancy as has been initially described in bacteria (Segall et al., 1986; Sourjik and Berg, 2002). On the other hand, high-order receptor assemblies may occur. For example, a supramolecular activation cluster can occur in mammalian cells. This is the case of multiple T-cell receptors that may complex with MHC–peptide antigens on the surface of a neighboring antigen-presenting cell (Krummel and Davis, 2002). These high-order receptor assemblies bring functional consequences (i.e., inter-receptor communication), increasing the system information processing capacity.

The evolutionary history of GPCR multi-protein complexes, including receptors, transduction systems and signaling modulators, is very well described in the work by de Mendoza et al. (2014). Their results suggest that the main elements composing the GPCR system are present since very ancient times (even before the appearance of the so-called common ancestor of eukaryotes, LECA) and characterized by independent, modular and conserved evolution. Additionally, the results suggest that LECA expressed a complex repertoire of GPCRs and that those expansions of the GPCR signaling system occurred in an independent way and -initially-, mostly in unicellular or colonial species. Their conclusion is that their results support the view that unicellular lifestyles also require complex signaling machineries (Crespi, 2001). On the other hand, receptor diversity explosion, originated concomitant with transition to multicellularity, and as previously mentioned, in a parallel way.

Beyond the molecular architecture diversity of GPCRs and associated multi-protein complexes, there are proteins that interact with some of them, the so-called receptor activity-modifying proteins (RAMPs) that modulate GPCRs function and are important for our present discussion. RAMPs are unique proteins that upon binding GPCRs change their “shape”, and can act as pharmacological switches, or chaperones, that regulate signaling and/or trafficking in a receptor-dependent manner (Hay and Pioszak, 2016). In mammals RAMPs constitute a small family of three proteins able to introduce functional diversity by interacting with GPCRs (Smith and Scott, 2002). They were initially identified as chaperones that enhanced cell surface expression of the calcitonin-like receptor (CLR, historically known as CRLR) (Nair et al., 2019). Nowadays, RAMPs interactions with GPCRs provide an elegant mean for controlling GPCRs function that may provide further opportunities for drug development.

As summarized by Hay and collaborators (2016), four major roles have been reported for RAMPs: (1) enable the forward-trafficking of some GPCRs to the cell surface; (2) can alter GPCR pharmacology, switching ligand selectivity for some GPCRs; (3) can influence coupling to GPCR signaling pathways; and (4) may alter the trafficking pathway of some GPCRs from the cell surface, with different RAMPs controlling receptor fate through recycling or degradative pathways (Hai et al., 2016).

Structurally, vertebrate RAMPs comprise a single transmembrane spanning domain, an extracellular N-terminal domain of ~90–100 amino acids (RAMP2 having the longer sequence) and a short intracellular C-terminal domain of ~9 amino acids. Although potential glycosylation sites are present in some vertebrate RAMP1, mammalian RAMP1 appears not to be glycosylated. However, RAMP2 and RAMP3 have multiple glycosylation sites suggesting that this molecular modification may be fundamental for receptor function (Smith and Scott, 2002).

**Figure 2:**
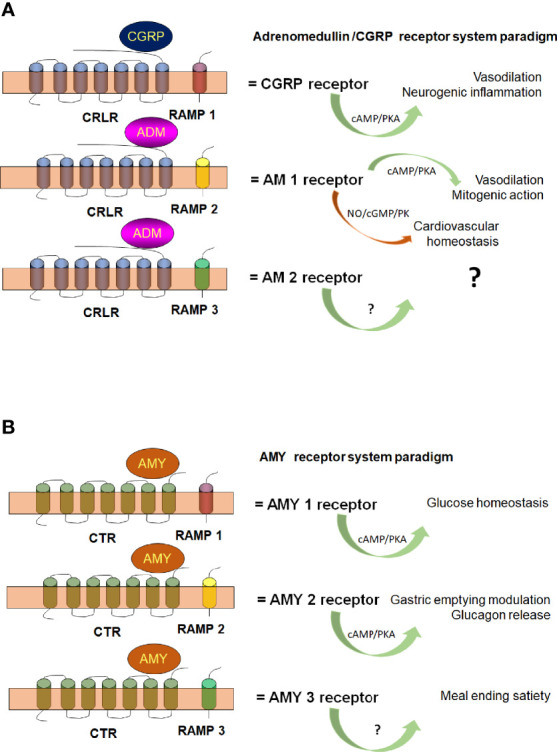
Hypothetical schematic diagram of pluricellular eukaryote (mammalian) Calcitonin Receptor Like Receptor [CRLR **(A)**] and Calcitonin Receptor [CTR **(B)**]. The scheme illustrates CRLR and CTR association with RAMP-1 (red), RAMP-2 (yellow) and RAMP-3 (green) to conform the different G protein receptors subtypes and their functions. AM, adrenomedullin; AMY, amylin; cAMP, cyclic AMP; cGMP, cyclic GMP; CGRP, calcitonin gene related peptide; NO, nitric oxide; PK, protein kinase. With permission of Elsevier (Febres et al., 2018; Febres et al., 2021).

Interactions of vertebrate RAMPs with class B-GPCR (CRLR), are the most extensively studied (Pioszak and Hay, 2020). RAMPs are required to guide CRLR-receptor to the cell surface, where RAMP/CRLR complexes act as receptors for peptide hormones such as the calcitonin gene-related peptide (CGRP) or adrenomedullin (AM), depending on RAMP co-expression (Heldin, 1995). CRLR/RAMP1 conform the CGRP receptor and CRLR/RAMP2 or CRLR/RAMP3 conform the AM1 or AM2 receptors, respectively (Falke and Hazelbauer, 2001). RAMPs seem to interact with other GPCRs, although as mentioned, RAMPs properties have only been extensively studied for CRLR or calcitonin receptors (CTR). **Figure 2** illustrates a hypothetical schematic diagram of pluricellular eukaryote (mammals) interaction of RAMPs with GPCRs.

## Transduction Systems, Intracellular Messages

We already mentioned that upon ligand binding, the targeted cell surface receptor experiences a conformational change that ends up in the activation of its cytoplasmic transducers through specific mechanical processes that characterize each receptor type (Nair et al., 2019). For GPCRs, ligand binding promoted conformational changes activate associated heteromeric G-proteins. This is mediated by the binding of guanosine triphosphate (GTP) (Trzaskowski et al., 2012). Activated G-proteins dissociate into Gα and Gβγ subunits (activators of diverse second messengers, channels, kinases, phospholipase, and adenylyl cyclase isoforms) (Dascal, 1997; Hanoune and Defer, 2001; Siehler, 2009; Vanhaesebroeck et al., 2012; Kadamur and Ross, 2013; Lyon and Tesmer, 2013; Zamponi and Currie, 2013).

GPCRs may also function independently of G-proteins *via* the G protein-coupled receptor kinase (GRKs), β-arrestin (Ribas et al., 2007), and non-receptor tyrosine kinases Srcs (Cao et al., 2000). Additionally, they can activate monomeric small GTPases, namely Ras (Zenonos and Kyprianou, 2013), like Ras-homologous (Rho) protein (Schwartz, 2004), Ras-associated binding (Rab) proteins (Schwartz et al., 2007), ADP ribosylation factor (Arf) (Jackson and Bouvet, 2014), and Ras-related nuclear protein (Ran) (Caputo et al., 2015). These small GTPases act as central transducers for diverse signaling pathways. Finally, guanine nucleotide exchange factors (GEFs) can activate the GTPase through the replacement of guanosine diphosphate (GDP)/GTP. Even a system approach has been designed to investigate GPCR-mediated Ras signaling network in chemoattractant sensing in Dictyostelium cells by combining live-cell imaging and computation models. By this approach, the authors validated the dynamics of signaling events and even predicted dynamic profiles of receptor-mediated Ras signaling networks including defective Ras inhibitory mechanisms (Xu et al., 2022).

Activated GTPases interact with specific downstream effectors to sequentially trigger the activation of particular effectors to generate diverse second messengers; i.e., small dedicated molecules like cAMP, cGMP, IP_3_, diacylglycerol, calcium, etc., whose intracellular levels are critical and should be strictly regulated to guarantee cell homeostasis.

Understanding the sensory and effector behavioral responses of cells to environmental signals, and how they interact to preserve cell homeostasis and performance, are fascinating themes that can be analyzed by means of cell physiology and genomic studies. The same is true for the comprehension of the gene organization and expression that influence cell signaling, as well as for the molecular relationships that exist between signaling intermediates (Lauffenburger, 2000; Needham et al., 2019).

On the other hand, experimental and computational integration of data is a fundamental mean to develop signaling network models (Dubitzky et al., 2005). Its comprehension may be useful in the understanding of inter- cellular and organellar (compartmentalized) signaling development (Wang and Zhang, 2012).

Additionally, biochemical and biophysical techniques containing structural and dynamic information are key for the understanding of the specific activation and signal transduction of GPCRs and to define the receptor–ligand interface, the conformational changes of both ligand and receptor during the binding process, and the structural plasticity of the receptor itself (Kaiser and Coin, 2020).

These techniques include for example, mutagenesis and crosslinking as well as spectroscopic techniques like nuclear magnetic resonance (NMR), electron paramagnetic resonance (EPR), fluorescence and Fourier-transform infrared spectroscopy (FTIR), among others.

We will focus on what has been described for Trypanosomatidae, especially for *Leishmania*, our model of study.

## G Protein Signaling Mechanisms in Trypanosomatidae

In Trypanosomatidae parasites, unique mechanisms of cyclic nucleotide signaling have been described. In fact, cyclic nucleotides seem to be essential for parasitic proliferation and differentiation. For example, since the nineties, life-stage specific functional elements of G-proteins have been demonstrated in *Trypanosoma (brucei) brucei* (Coulter and Hide, 1995) as well as in *Trypanosoma cruzi* (Oz et al., 1994), however, canonical G-proteins have not been still recognized and seem to be absent at least in the genome; bioinformatic analysis yields data for encoding GPCRs that seem to be without functional proof, and small GTPases or their secondary effector proteins with structural differences to host orthologues seem to constitute the molecular mean for the role cyclic nucleotides have (Kaiser, 2019).

Despite the apparent absence of subunits of canonical G-proteins and the presence of key components of these signal pathways, GPCR-mediated signaling research has been stimulated by the good news of the completion of Trypanosomatidae parasites genome sequences (Hamm, 1998). Of note, some database entries highlight encoding proteins with serpentine motifs that might have GPCR-like functions as will be mentioned afterwards.

Very recently, the group of Keith Matthews has identified a molecule, part of the GPR89-family of proteins, located at the *Trypanosoma (b.) brucei* parasite surface that regulates differentiation to stumpy forms. TbGPR89 with oligopeptide transport capabilities, is expressed on bloodstream ‘‘slender form’’ trypanosomes; when ectopically expressed, TbGPR89 drives stumpy formation in a *Stumpy Inducing Factor* (SIF)-dependent process. As mentioned in the discussion of their article “collectively, these data provide a ‘‘signal’’ and ‘‘receptor’’ mechanism for density sensing in trypanosome infections” (Rojas et al., 2019). Plant members of the GPR89 family (GTG1 and GTG2) are considered orphan GPCRs (Pandey et al., 2009). This means that TbGPR89 constitutes the first demonstration of an orphan G protein-coupled receptor related protein in Trypanosomatidae.

On the other hand, cAMP is a fundamental second messenger molecule in Trypanosomatidae; more than 80 genes and pseudogenes encoding adenylate cyclase (ACs) have been reported in the *Trypanosome* genome and descriptions of adenylate cyclase X-ray structure from *T. (b.) brucei* (Jansen et al., 2013) have been published. *T. (b.) brucei* AC belongs to the type II class of ACs of protozoa; it has only one transmembrane domain as well as a large variable extracellular domain -for putative receptor binding-, while their counterparts in mammals have 12 transmembrane domains. Additionally, both the mammalian and *Trypanosome* AC seems to share a basic catalytic mechanism and displays a cytosolic, catalytic region (Gould and de Koning, 2011). Of note, cAMP/AMP might have a role in stressful conditions such as quorum-sensing signaling in *T. (b.) brucei*, as well as the downstream cAMP Response Proteins (CARPs), whose expression levels correlate with sensitivity to phosphodiesterases (PDEs) inhibitors (Tagoe et al., 2015)

Dimerization of AC has been described in *T. cruzi*. The *T. cruzi* AC seems to participate in metacyclogenesis [the conversion of epimastigotes in the insect midgut, and later in the hindgut, into human-infectious non-proliferative metacyclic trypomastigotes] (Gonzáles-Perdomo et al., 1988; Fraidenraich et al., 1993; García et al., 1995). Even more, *in vitro*, nutritional stress induced metacyclogenesis causes an increase in cAMP production and levels, thus suggesting that this might be prerequisite for their final differentiation to metacyclic trypomastigotes (Boker and Schaub, 1984). All these data suggest that AC signaling might constitute a complex cascade, especially since as it has been demonstrated, Trypanosomatidae PDEs are highly conserved in these parasites although almost insensitive to mammalian inhibitors (Tagoe et al., 2015; Kaiser, 2019).

Small GTPases [most of them belonging to the Ras superfamily (Rojas et al., 2012)] are also expressed in Trypanosomatidae. These small GTPases act as molecular switches and seem to be fundamental for successful host infection in the absence of canonical G-proteins and putative GPCRs. Functions like the control of gene expression and cell proliferation, vesicle coating, nucleo-cytoplasmatic transport and regulation of cell cycle progression need the action of the Ras superfamily. Trypanosomatidae express Rab (Ras related proteins in brain) proteins controlling intracellular, as well as vesicular transport and Rho GTPases that participate in host pathogen interaction and control innate and adaptive immune responses (Thumkeo et al., 2013). In fact, Rab5 isoforms are related to endocytic functions in *L.* (*L.*) *donovani* upregulating their expression in early endosomes (Verma et al., 2017), and regulating fluid-phase endocytosis (Rastogi et al., 2016) in these parasites.

Interestingly, stimuli initiated in parasites can trigger GPCR-mediated signaling in host cells during the process of parasite invasion and egression (Kaiser, 2019). In fact, the macrophage transmembrane costimulatory receptor CD40, plays an important role in *Leishmania* infection. For example, during initial steps *Leishmania* infection relies on parasite-expressed lipophosphoglycan (LPG) interaction with the host cell-expressed TLR-2, this not necessarily being a host-protective response. In fact, interactions between LPG and TLR-2 reduce anti-leishmanial responses *via* cytokine-mediated decrease of TLR-9 expression (Srivastava et al., 2013). Of note, additional TLRs are differentially involved in infections by other *Leishmania* species (Faria et al., 2012; Sacramento et al., 2017), suggesting that TLRs participation may lead the activation of adaptive immune responses, CD40 playing a crucial role, as its expression may be enhanced by pathogen-derived nucleic acids that recognize intracellular TLRs. This up-regulation of CD40 by intracellular TLR can then represent a host-protective strategy, as CD40 signaling may activate downstream signaling intermediates such as MAPK, PKC and Ras. These results suggest that the parasite may modulate the use of the available signaling to decide the downstream signaling pathways and signaling molecules (Chandel et al., 2014).

In this regard, CD40 induces activation of different Ras-isoforms. As the receptor has dual signaling functions, it can stimulate either Raf-MEK-ERK-1/2-mediated anti-inflammatory IL-10 production or P13K-MKK-p38MAPK-mediated proinflammatory IL-12 production (Chakraborty et al., 2015). Thus, depending on the Ras isoform used, the outcome of a *Leishmania (L.) major* infection can differ due to the capacity of the parasite to switch the signal. CD40-induced IL-10 promotes *Leishmania* infection whereas CD40-induced IL-12 protects hosts from infection (Chakraborty et al., 2015). This is also true for *L.* (*L*.) *donovani*. Infection with these parasites leads to an increased expression of N‐Ras, whereas the expression of K‐Ras and H‐Ras decreases. The increased expression of N-Ras leads to a tight regulation of phosphorylation/dephosphorylation cycles of extracellular signals that may lead to the regulation of interleukin-10 and -12 secretion, suggesting that in *L. (L.) donovani* N-Ras expression may be associated to a novel immune evasion strategy (Husein et al., 2018)

In *T. cruzi*, a Ras-related GTPase mainly found in the GTP-bound stage, is localized closely to the flagellar apparatus (dos Santos et al., 2012). Its function is required for cell growth and its overexpression blocks *T. cruzi* metacyclogenesis. Additionally, the Rho GTPase Rac1, is fundamental for a host cell actin-dependent invasion by amastigotes of *T. cruzi* (Bonfim-Melo et al., 2018).

In *Leishmania* a variety of GTPases with functions in intracellular trafficking, secretory pathways, endocytosis and pathogenesis exist. In fact, a monomeric GTPase Rab1 homologue has been characterized in the secretory pathway of *Leishmania* (Bahl et al., 2015). On the other hand, trafficking and secretion of gp63, the *Leishmania* virulence factor, is regulated by the GTPase Sar1 (Parashar and Mukhopadhyay, 2017) and is essential for parasite survival. Interestingly, Rab5 isoforms from *Leishmania* regulate fluid-phase or receptor-mediated endocytosis (Rastogi et al., 2016).

So, the conclusions to be driven from these examples are: (1) although the information in Trypanosomatidae is mainly related to the presence and function of small GTPases, the existence of canonical G-proteins (or succedanea) cannot be ruled out, as could be deduced from the existence of a putative guanine nucleotide-binding protein subunit alpha reported in *L. (L.) donovani* (UniProtKB code P43151), in addition to three G protein gamma domain-containing proteins found in *L. (L.) donovani* (strain BPK282A1), *Leishmania (L.) mexicana* (strain MHOM/GT/2001/U1103) and *L. (L.) major* (UniProtKB codes E9BTQ7, E9ASW2 and Q4Q1N4, respectively) [The UniProt Consortium. UniProt: the universal protein knowledgebase in 2021. Nucleic Acids Res. 49: D1 (2021)]; and (2) that canonical cyclic nucleotide pathways expressed in the host are thoroughly used by the parasite to promote pathogenicity. The next chapter will focus on further evidences of their presence in *Leishmania.*

## GPCR_LIKE_ Signaling Systems in Leishmania

*Leishmania* parasites are digenetic. They oscillate between a mammalian and an insect vector host, thus exhibiting a complex life cycle with developmental stages that represent an everyday challenge for these organisms (see **Figure 1**). Differentiation into the next developmental stage implies the evolution of mechanisms and systems that must help the parasite to sense and respond to the microenvironment imposed by the host. This is a key step in their survival since they must adapt their biology and physiology to the immediate *milieu* and therefore should have the capacity to detect and respond to the external oscillations (Rotureau et al., 2009). What kind of sensory systems they express, which kind of signals they detect and how they integrate the received information into a behavior, are key questions to be analyzed. For example, parasite differentiation has been demonstrated to be a prerequisite for successful vector infection, vector–host transmission and propagation in *T. (b.) brucei* (Fenn and Matthews, 2007). Thus, the comprehension of these issues is fundamental to understand *Leishmania* virulence and pathogenesis. In fact, very recent work has focused on the influence of resident microbiota on vector infection by *Leishmania*, describing that the sand fly microbiota is central for *Leishmania* development and transmission. Even it has been suggested that the removal of microbiota alters the osmolarity of the intestinal environment and is deleterious for *Leishmania* development (Telleria et al., 2018).

On the other hand, the inoculation of (metacyclic) promastigotes into the skin (human or mammalian reservoirs) by the vector bite initiates the infection, meaning an immediate change in the environment. Invading parasites are recognized as alien agents, trigger complex physiological responses, and guide host actions designed to isolate and eliminate the invading pathogen present in the skin. The response includes activation of inflammatory and immunological cells and molecules, among them neuropeptides, cytokines, etc., that modulate and impact the final result. Would it be disease installation (with parasite survival)? or (if the host can control the infection) variable rates of spontaneous healing? (Díaz and Ponte-Sucre, 2018). *Leishmania* parasites and the cutaneous nervous system roles for successful macrophage-promastigote interaction and initiation of inflammatory processes are pivotal to have one or other result (Giammarressi et al., 2020). The final answer will depend on the response to the received signals, the skin being a key place where these signals are processed to build up a reply, since the skin as an organ, reacts to endogenous and exogenous signals, perceive and integrate environmental stimuli, and reacts.

In this process, *Leishmania* membrane proteins accomplish fundamental roles, including the primary function of sensing minimal changes in their *milieu* and working as a communication interface between extracellular and intracellular settings. The aim, to preserve integrity and survival of the parasite through its life cycle including host invasion. Additionally, skin biological active peptides are released during *Leishmania* infection. These molecules modulate host-parasite interaction and play key roles in the end result of the infection.

*Leishmania* models have been used to evaluate the intimate link between the parasite and its milieu, the sensory and autonomic neuropeptides, and macrophage function during parasite infection (Ahmed et al., 1998a; Ahmed et al., 1998b; Ahmed et al., 1998c; Ahmed et al., 2001).

In our research work and to comprehend the parasite physiological behavior in such stressful surroundings, we initially began to dissect which molecules might be involved in the migratory responses of *Leishmania*. Originally, we assayed molecules which constitute main energy sources for the parasite (carbohydrates, amino acids) crucial for their differentiation and motility in the vector (Díaz et al., 2015; Díaz et al., 2021). These initial results and the chemotactic responsiveness obtained suggest that *Leishmania* discriminate between slight differences in concentration of small and structurally closely related molecules and indicate that besides their metabolic effects, amino acids (AAs) play key roles linked to sensory mechanisms that might determine the parasite’s behavior (Díaz et al., 2015). These responses might involve signal transduction systems and receptor types not yet described.

Moreover, both sensory (substance P, SP; calcitonin gene-related peptide, CGRP; and somatostatin, SOM), and autonomic (neuropeptide Y, NPY; and vasoactive intestinal peptide, VIP) neuropeptides, have been used in *L. (L.) major*-macrophage models to analyze macrophage function (Ahmed et al., 1998a; Ahmed et al., 1998b; Ahmed et al., 2001). The results indicate that neuropeptides modulate the initial steps of host-parasite interaction and influence disease development by affecting promastigote-induced macrophage migration. Besides, in both BALB/c and C57BL/6 models of infected mice, CGRP and NPY skin concentrations decrease significantly throughout the study period as compared with control animals. Of note, CGRP concentrations were about 4, 8 and 9 times lower, respectively at 3-, 6- and 9-weeks post-infection, in the skin of infected BALB/c mice compared with infected C57BL/6 skin (Ahmed et al., 1998b; Ahmed et al., 1998c). Finally, parasite killing can be caused by high doses of VIP (Campos-Salinas et al., 2014).

Whether or not the effect of these neuropeptides is mediated by *Leishmania* GPCRs has not been elucidated. As mentioned, canonical G-protein signaling pathways have not been defined in members of the Trypanosomatidae. Nevertheless, two putative extracellular receptors with possible G protein-coupled GABA receptor activity have been described in the genomes of *L. (L.) major* and *Leishmania tarentolae* (*Sauroleishmania tarentolae*) (UniprotKB codes Q4QDF7 and A0A640KFI9, respectively).

GPCRs are flexible in their molecular architecture, including RAMPs proteins common among multicellular eukaryotes, as modulators that interact with GPCRs to modify their function. However, RAMPs expression has not yet been reported in unicellular eukaryotes; their description in these organisms can be crucial, since pharmacological intervention of conserved G protein signaling pathways might be helpful to dissect the physiology of eukaryote pathogens and RAMPs constitute ideal targets for tool design against diseases caused by them.

To contribute to the GPCRs portrayal as a potential fundamental signaling system in leishmaniasis pathophysiology, we have evaluated the migration of parasites in a controlled system in the presence of some of these neuropeptides. **Table 1** summarizes the chemotactic effect produced by selected neuropeptides on the migration of *L. (V.) braziliensis.* The table includes the potential origin of these neuropeptides in mammals as well as the usually found associated accessory protein in the system. The data presented demonstrates the *in vitro* effect of sensory [Substance P, SP (10^-8^ M), chemoattractant] and autonomic [(Vasoactive Intestinal Peptide, VIP (10^-10^ M), and Neuropeptide Y, NPY (10^-9^ M), chemorepellent] neuropeptides at physiological levels on parasite taxis. And additionally, the chemorepellent effect of the vasoactive molecules Calcitonin Gene-Related Peptide [(CGRP) (10^-9^ and 10^-8^ M)] and Adrenomedullin [(AM) (10^-9^ to 10^-5^ M)] (Febres et al., 2018; Giammarressi et al., 2020; Febres et al., 2021).

**Table 1:** Chemotactic effect of selected neuropeptides in L. (V.) braziliensis.

	Effect in *L. (V.) braziliensis*		Functional properties in mammals		
Neuropeptide^1^	Chemorepellent ^2^	Chemoattractant ^3^	Type ^4^	Associated RAMP ^5^	References
CGRP	10^-9^ - 10^-8^ M	–	Sensorial	RAMP1	(Peters et al., 2006; Hay and Pioszak, 2016)
AM	10^-9^ - 10^-5^ M	–	Sensorial	RAMP2, RAMP3	(Peters et al., 2006; Hay and Pioszak, 2016)
VIP	10^-10^ M	–	Sympathetic	RAMP2	(Couvineau and Laburthe, 2012)
NPY	10^-10^ - 10^-9^ M	–	Sympathetic	nd	(Holzer et al., 2012; Sheng and Zhu, 2018)
SP	–	10^-9^ - 10^-7^ M	Sensorial	nd	(Vidal Yucha et al., 2019)

nd, not determined. ^1^ CGRP, Calcitonin Gene Related Peptide; AM, Adreno medullin; VIP, Vasoactive Intestinal Peptide; NPY, Neuro peptideY; SP, Substance P. ^2,3^ Neuropeptide concentration that exerts a significative effect in L. (V.) braziliensis. ^4^ Mammalian nervous system origin of the neuropeptide. ^5^Accessory protein described to be associated in mammals.Functional properties in mammals.

**Figure 3:**
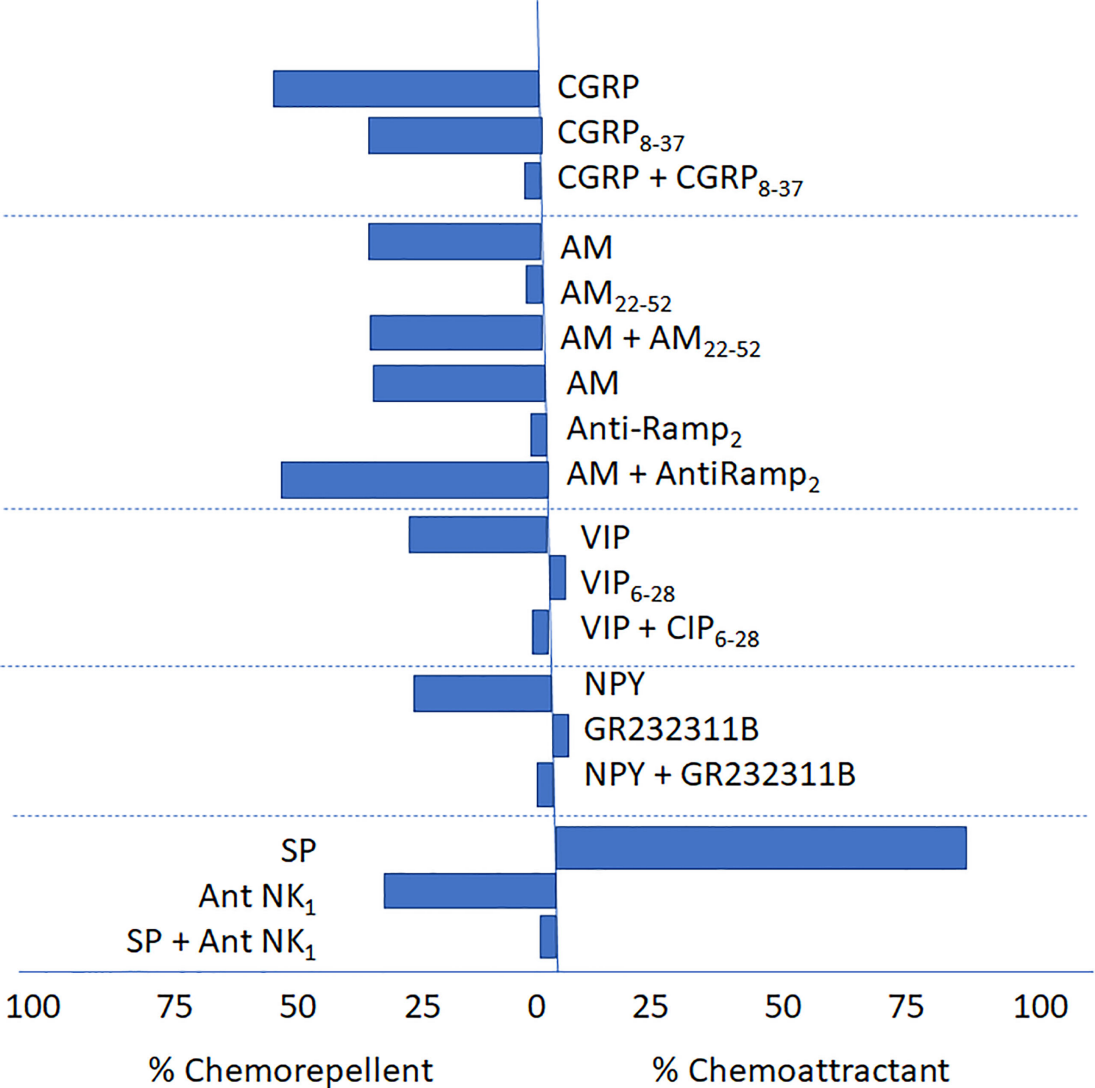
Antagonism of CGRP8–37, AM22–52, Anti-RAMP-2, VIP (VIP6-28), NPY (GR2323118) and (D-Pro2, D-Trp7,9]-Substance P, on the chemotactic response elicited by CGRP, AM, VIP, NPY and SP, on *L. (V.) braziliensis*. (Febres et al., 2018, Giammarressi et al., 2020; Febres et al., 2021). The figure details the chemoattractant or chemorepellent effect (as percentages, see the abscisa) of sensorial and autonomic peptides and their respective antagonist (either alone or in combination with the agonist) and their effect on the migratory response in *Leishmania* (*V*.) *braziliensis.* In the case of AM a specific antibody, Anti-RAMP-2 was also included.

We have also demonstrated, as observed in **Figure 3**, that VIP and NPY chemotactic effects are impaired by their corresponding receptor antagonists and that the effect of SP is blocked by ([D-Pro 2, D-Trp7,9]-Substance P (10^-6^ M), suggesting that it might be mediated by neurokinin-1 transmembrane receptors.

Similarly, the chemorepellent effect of the vasoactive molecule CGRP at physiological levels was specifically blocked by its corresponding antagonist CGRP_8-37_; however, the chemorepellent effect of AM was not blocked by AM_22-52_. even at concentrations 100 to 1000-fold higher than those used to elicit the chemotactic effect. Of note, CGRP_8-37_ by itself but not AM_22-52_, produce a chemorepellent effect. Altogether these results suggest the presence of GPCR receptors or GPCR-like receptor signaling system in *Leishmania* not homolog to those expressed in vertebrates (Febres et al., 2018; Giammarressi et al., 2020; Febres et al., 2021).

## RAMP or RAMP-Like Proteins in Leishmania

The presence of an antigenically related 24 kDa peptide (identified with human RAMP-2 antibodies) associated with RAMP or RAMP-like proteins was further demonstrated by western blot analysis in *Leishmania* sp. homogenates of the strains *Leishmania* (*L*.) *amazonensis* (LTB0016), *L*. (*L*.) *mexicana* (Bel21) and *L*. (*V*.) *braziliensis* (LTB300) (Febres et al., 2018; Febres et al., 2021). Western blot analysis also confirmed the expression of this band in *Leishmania* isolates [*L*. (*L*.) *mexicana* and *L*. (*L*.) *amazonensis* respectively, (VE96ZC; VE98MR, and VE2000MM)] isolated from patients suffering active Diffuse Cutaneous Leishmaniasis with therapeutic failure against Glucantime^®^. This data confirms in many *Leishmania* strains and species the presence of at least peptides that could be recognized by RAMP-2 specific antibodies, suggesting the presence of the related proteins.

**Figure 4:**
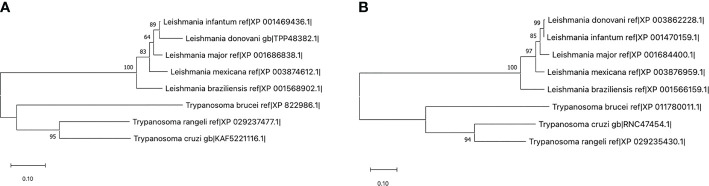
Maximum likelihood trees using the sequences for *Leishmania (V.) braziliensis* XP_001568902 **(A)** and XP_001566159.1 **(B)** in comparison with their respective homologs in other trypanosomatids. Those sequences with an identity percentage of at least 80% through DELTA-BLAST were selected for construction of the tree. Note that the proteins pertaining to the *Leishmania* genre lie in a different clade than those from *Trypanosoma*, and that other trypanosomatidae species including *Crithidia* and *Sauroleishmania* do not express a protein below the selected identity threshold (Febres et al., 2018; Febres et al., 2021).

Furthermore, when using the protein sequence of RAMP-2 human isoform against the predicted proteome of *L. (V.) braziliensis* by means of DELTA-BLAST, an alignment with the folylpolyglutamate synthase (FPGS), XP_001568902, of *Leishmania* was identified. A similar approach using the human RAMP-3 sequence detected an alignment against a hypothetical protein in *Leishmania* with yet unknown function, XP_001566159.1. The worth of these alignments was confirmed with prss3. Orthologs to these proteins were found in additional species of the *Trypanosomatidae* family, including species of *Leishmania* and *Trypanosoma* as can be seen in **Figure 4**. Notwithstanding, an ortholog to the protein was not found in more divergent species of the family, such as *Crithidia* and *Sauroleishmania* (Febres et al., 2018; Febres et al., 2021).

The presence of transmembrane helices in these proteins as predicted with Phyre2, suggests a role in either intercellular or intracellular signaling. Protein interaction networks of *Leishmania* species (dos Santos Vasconcelos et al., 2018) localizes 17 proteins of the *L. (V.) braziliensis* species to the plasma membrane, with 351 having a predicted functional compartment in the cytosol, but more evidence would be required to accurately identify the functional compartment of the proteins. Using Pfam, a Mur ligase homolog domain was encountered in the RAMP-2-aligned protein, suggesting a role in the regulation of the membrane rigidity, potentially translating into changes in the motility of the parasite (Febres et al., 2018; Febres et al., 2021).

**Figure 5:**
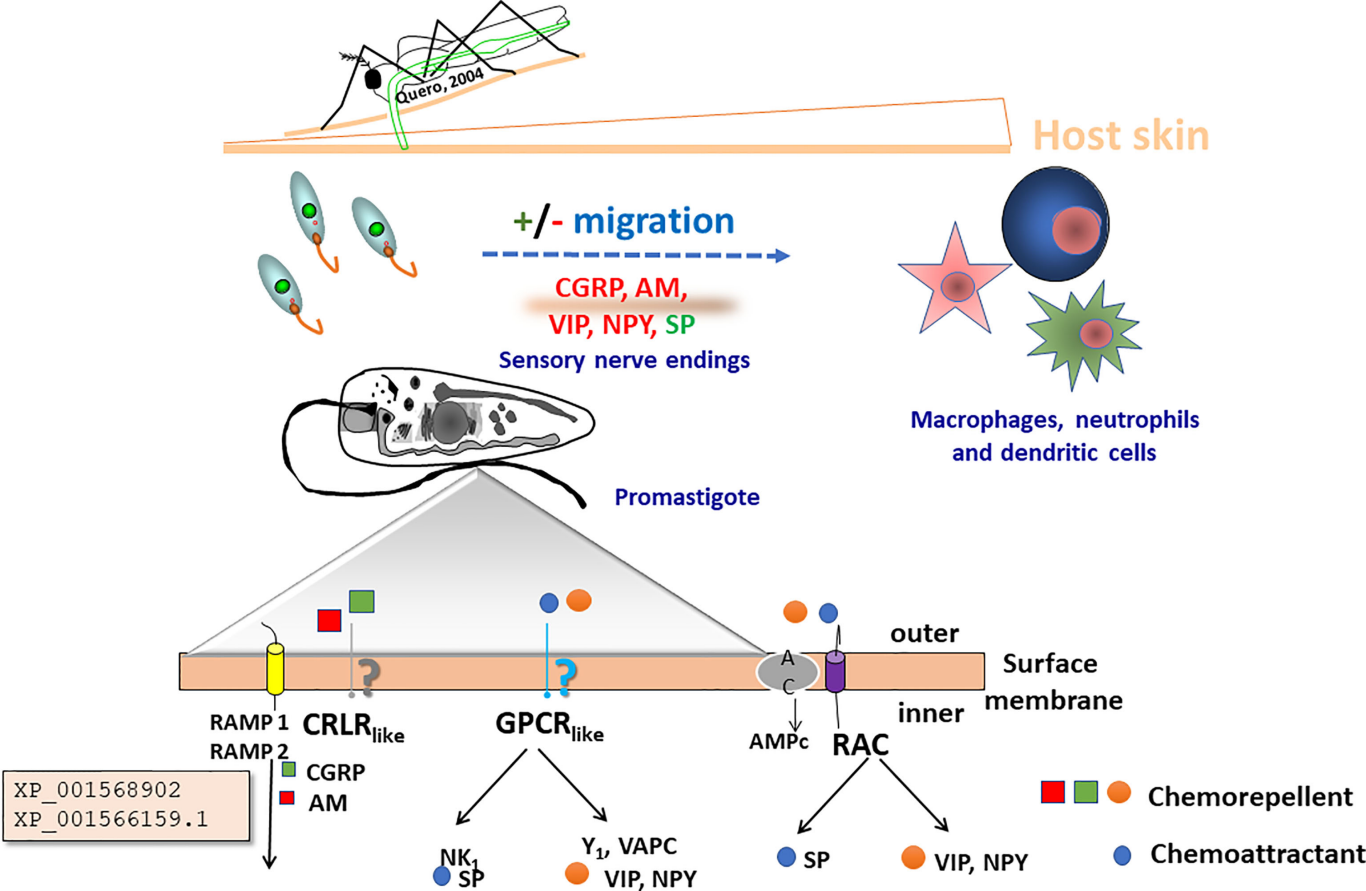
Proposed mechanism by which the novel *Leishmania* proteins, XP_001568902 and XP_001566159.1, may play a role in the completion of the lifecycle of the parasite. After first inoculation of the promastigote into the skin, the parasite in its promastigote form reacts against neuropeptides secreted by the nerve terminals in the skin. Adrenomedullin (AM) secreted by the epithelial cells, and calcitonin gene-related peptide (CGRP) induce a negative chemotactic response in the parasite. Potentially they may induce metabolic activation before the invasion of the macrophages and the dendritic cells (DC). The zoom detail depicts flagellum membrane organization including a scheme of potential transmembrane receptors that may be (NK1, Y1 and VAPC), G-protein (like), CRLR (like) coupled RAMP (1,2) receptors (Giammarressi et al., 2020), and RAC receptors.

Cellular switches like small G-proteins (Maheshwari et al., 2018) or cyclic adenosine monophosphate (cAMP)-dependent cellular transduction systems (Tagoe et al., 2015) are described to play a role in the biology of *Leishmania*, although the details regarding the precise mechanisms that orchestrate the kinetics of these messengers are poorly understood. *Leishmania* expresses isoforms of the ACs with a molecular structure that differs to that of superior eukaryotes (Sánchez et al., 1995; Kelly et al., 2021). Whether the proteins herein described relate to these mechanisms remain an unanswered question, but their conservation among the different species of *Leishmania* (*Viannia)* and *Leishmania (Leishmania)* indicate a fundamental role for cell survival. Our data also suggest that CGRP and AM levels in the *milieu* may modulate, through homologs of RAMP- (-2) and (-3), the taxis behavior of the parasite as presented in **Figure 5**.

## Final Remarks

*Leishmania* digenetic style of life cycle imposes that, migratory responses guided by chemotaxis may be key steps for *Leishmania* pathogenesis. Thus, the comprehension of which chemical signals are involved in the in-between host and parasite recognition is determinant for the fate of infection; the same is true for the identification of molecules, signs and behaviors involved in the responses (Díaz and Ponte-Sucre, 2018).

The analysis of chemotaxis may constitute an initial approach for the detection of molecules or drugs affecting parasite migration. Our proposal is its use as a preliminary step for discrimination of compounds to counteract *Leishmania* infection. Migration permits the evaluation of taxis (chemoattractant or chemorepellent) to discriminate whether or not responses are interesting to dissect (Díaz et al., 2011).

Thus, herein we propose (see **Figure 5**) *Leishmania* as a cellular model, with flagellar membrane receptor diversity, in which neuropeptide derivatives or designed molecules, i.e., conjugated with methotrexate (Díaz et al., 2013), could act to intervene the initial phase of *Leishmania* infection.

As herein described, we have tackled neuropeptides usually liberated in the skin once it is perturbed. These molecules should exert their stimulatory or inhibitory effect at physiological concentrations. In fact, in our experiments, physiological concentrations of these molecules triggered chemotactic responses in *Leishmania* and could influence skin invasion.

The autonomic neuropeptides VIP and NPY triggered “an escape response” on promastigotes that “swim” away from the stimuli; the same happened with CGRP and AM (Febres et al., 2018; Giammarressi et al., 2020; Febres et al., 2021). The take home message seems to be that as parasites and host-cells distance from each other, a “potential protection” of the host against infection develop. VIP and NPY decreased the percentage of infected macrophages in a *L. (L.) major* model, supporting the herein presented data and also indicating an effect of these neuropeptides also on phagocytosis (Ahmed et al., 2001).

Conversely, SP was chemoattractant to *L. (V.) braziliensis* promastigotes, increasing parasite migration, although decreased the percentage of parasites adhered to the macrophage surface (Giammarressi et al., 2020). More evidence is needed to support that this is a highly conserved chemotactic signaling mechanism related to the fact that the vertebrate signal molecules (e.g., neuropeptide) concentrations needed for optimal chemotactic effect in *Leishmania* or in *Tetrahymena* are very similar to the concentration (range 10^-12^ - 10^-9^ M) found in the vertebrate/human circulatory system (Köhidai and Csaba, 1996; Febres et al., 2018; Giammarressi et al., 2020; Febres et al., 2021).

Autonomic nerve terminals (innervating vascular smooth muscles and located in the skin interface) may release vasoactive peptides producing vasodilation upon insect bite, facilitating the arrival of macrophages to the site of infection. Alternatively, they may act maintaining promastigotes on the skin, preventing them from entering the systemic circulation due to a chemorepellent effect as herein described and previously demonstrated by Ahmed et al. (1998a; 1998b). VIP may be one neuropeptide acting as endogenous regulator of the immune homeostasis; the physiological consequences of its presence in the immune microenvironment depending on the timing of neuropeptide release and the activation stage of the neighboring immune cells (Ganea and Delgado, 2001).

Alternatively, the relative concentration of different peptides released by sensory nerve terminals could damage the sensory terminals if SP predominates, due to its chemoattractant effect, or alternatively -in vectors- this chemoattractant effect could be decisive for the migration of the parasite towards the salivary glands. Tackinins and the functions they may exert in invertebrates have been described. A few interesting cases exist where invertebrate tackinins are injected into prey animals as vasodilators from salivary glands, or may act as paralyzing agents from venom glands. The peptides are produced in the glands of predators; the sequences mimicking the prey tackinins (Nässel et al., 2019). This may well be happening in the case of *Leishmania.*

What is clear is that our data provides evidence that vascular, sensory and autonomic neuropeptides exert modulating effects on parasite migration, suggesting a potential role neuropeptide could have on host-parasite interaction, modulating the natural course of the protozoan life cycle during its stressful traveling between both hosts and within the host cell.

The findings suggest that proteins and molecules potentially involved in the associated receptor cascade, with similar functions to those present in higher eukaryotes, signpost conservation of ancient signaling systems associated with unicellular responses, fundamental for cell survival, i.e., taxis and migration (Kennedy, 2013). Further research is needed to elucidate whether or not GPCR receptors and their potential associated proteins are involved in the intrinsic mechanisms of the herein described effects, especially for SP (**Figure 5**). This neuropeptide could be acting through a different process than the chemorepellent peptides. Of note, it seems to be important to be open mind and better characterize the so-called group of orphan receptors, especially those belonging to GPCRs repertoire that have been described to have no known function. Might it be possible that they conserve the functional characteristics of GPCR in spite of being structurally different? Might it be that they comprise a GPCR-receptor-RAMP complex? Additional data are needed to clarify these queries.

On the other hand, we must mention that putative adenylate cyclase associated receptors (RAC) might also be linked with the neuropeptide associated chemotactic responses observed herein. These catalytic (enzyme) receptors modulate intracellular phosphorylation and levels of second messengers. Eleven genes that share structural features with those encoded by *Trypanosoma (b.) brucei* and *Trypanosoma equiperdum* have been described in *Leishmania*: an extracellularly N-terminal, a transmembrane domain and a catalytic C-terminal that associates with intracellular enzymes (Sánchez et al., 1995; Giammarressi et al., 2020).

In these parasites, genes encoding ACs have undergone considerable expansion and diversification (Durante et al., 2020) although the evolutionary forces shaping this phenomenon are poorly understood. The analysis of kinetoplastid ACs demonstrate a correlation with AC gene family expansion and lifestyle, these proteins potentially mediating immune evasion, insect tissue navigation, or even interaction with endosymbionts (Durante et al., 2020). However, a direct role for cAMP in the initiation of trypanosome differentiation events has become doubtful, but the role and importance of cAMP in flagellar motility and signaling is increasingly being dissected, with interesting findings. For example, it is commonly believed that the flagellum, as an important host-parasite interface, has essential sensory functions (Tetley and Vickerman, 1985; Rotureau et al., 2009; Tagoe et al., 2015; Kelly et al., 2021).

Finally, since the inhibition of flagellar motility has been proposed as a target for the study of new drugs against African trypanosomes (Broadhead et al., 2006), the study of chemotaxis in different strains of *Leishmania* would be very useful in phenotypes which should express differences in migratory responses. This would give way to a biochemical, cellular, proteomic and genetic approach, which should lead to an integrated view of the involvement of parasite motility during its life cycle, virulence and/or infectivity (Díaz et al., 2011; Díaz et al., 2021).

All in all, pharmacological intervention of conserved G protein signaling pathways may constitute targets for tool design against diseases caused by them. Their portrayal would be fundamental to provide sound patho-mechanistic concepts and novel therapeutic strategies for patients with the chronic, and frustrating to treat cutaneous leishmaniasis.

## Author Contributions

AF, MG, AS, and OV were responsible for the implementation of the methodology, investigation, validation and formal analysis of the data. Additionally, CG and AF were responsible for data curation, software and visualization, and review and editing. Furthermore, ED and AP-S were responsible for the conceptualization, supervision, project administration, funding acquisition, data curation, writing—review and editing and AP-S was responsible for the writing—original draft preparation. All authors have read and agreed to the published version of the manuscript.

## Funding

The authors are grateful to the Universidad Central de Venezuela Council for Research, grants CDCH-UCV PI-09-8717-2013/1 and PG-09-8646-2013/1.

## Conflict of Interest

The authors declare that the research was conducted in the absence of any commercial or financial relationships that could be construed as a potential conflict of interest.

## Publisher’s Note

All claims expressed in this article are solely those of the authors and do not necessarily represent those of their affiliated organizations, or those of the publisher, the editors and the reviewers. Any product that may be evaluated in this article, or claim that may be made by its manufacturer, is not guaranteed or endorsed by the publisher.
